# Blast overpressure induces shear-related injuries in the brain of rats exposed to a mild traumatic brain injury

**DOI:** 10.1186/2051-5960-1-51

**Published:** 2013-08-14

**Authors:** Miguel A Gama Sosa, Rita De Gasperi, Alejandro J Paulino, Paul E Pricop, Michael C Shaughness, Eric Maudlin-Jeronimo, Aaron A Hall, William G M Janssen, Frank J Yuk, Nathan P Dorr, Dara L Dickstein, Richard M McCarron, Mikulas Chavko, Patrick R Hof, Stephen T Ahlers, Gregory A Elder

**Affiliations:** 1 Department of Veterans Affairs Medical Center, General Medical Research Service, Bronx, New York, USA; 2 Department of Veterans Affairs Medical Center, Research and Development Service, Bronx, New York, USA; 3 Department of Veterans Affairs Medical Center, Neurology Service, Bronx, New York, USA; 4 Department of Psychiatry, Icahn School of Medicine at Mount Sinai, New York, New York, USA; 5 Fishberg Department of Neuroscience, Icahn School of Medicine at Mount Sinai, New York, New York, USA; 6 Department of Geriatrics and Palliative Care, Icahn School of Medicine at Mount Sinai, New York, New York, USA; 7 Department of Neurology, Icahn School of Medicine at Mount Sinai, New York, New York, USA; 8 Friedman Brain Institute, Icahn School of Medicine at Mount Sinai, New York, New York, USA; 9 Operational and Undersea Medicine Directorate, Naval Medical Research Center, Silver Spring, Maryland, USA

**Keywords:** Blast overpressure injury, Neuropathology, Shear injury, Traumatic brain injury

## Abstract

**Background:**

Blast-related traumatic brain injury (TBI) has been a significant cause of injury in the military operations of Iraq and Afghanistan, affecting as many as 10-20% of returning veterans. However, how blast waves affect the brain is poorly understood. To understand their effects, we analyzed the brains of rats exposed to single or multiple (three) 74.5 kPa blast exposures, conditions that mimic a mild TBI.

**Results:**

Rats were sacrificed 24 hours or between 4 and 10 months after exposure. Intraventricular hemorrhages were commonly observed after 24 hrs*.* A screen for neuropathology did not reveal any generalized histopathology. However, focal lesions resembling rips or tears in the tissue were found in many brains. These lesions disrupted cortical organization resulting in some cases in unusual tissue realignments. The lesions frequently appeared to follow the lines of penetrating cortical vessels and microhemorrhages were found within some but not most acute lesions.

**Conclusions:**

These lesions likely represent a type of shear injury that is unique to blast trauma. The observation that lesions often appeared to follow penetrating cortical vessels suggests a vascular mechanism of injury and that blood vessels may represent the fault lines along which the most damaging effect of the blast pressure is transmitted.

## Background

Traumatic brain injury (TBI) has been a common cause of mortality and morbidity in the military operations in Iraq and Afghanistan [1]. It is estimated that 10-20% of returning veterans have suffered a TBI [1]. Due to the prominent use of improvised explosive devices (IED) in Iraq and Afghanistan, a characteristic feature of TBI in these conflicts has been its association with blast exposure [2]. Single or multiple blast exposures have been commonly seen in association with chronic neurological and psychiatric sequelae including persistent cognitive impairment, post-traumatic stress disorder (PTSD) and depression [1]. Blast injuries occur through multiple mechanisms that may be related to effects of the primary blast wave, to injuries associated with objects including shrapnel contained within the IED being propelled by the blast wind, or by the individual being knocked down or thrown into solid objects [3].

How the primary blast wave itself affects the brain is not well understood [3]. Direct tissue damage, bleeding, and diffuse axonal injury (DAI) are the best known pathophysiological mechanisms associated with the type of non-blast TBI most commonly encountered during blunt impact injuries in civilian life [4,5]. Blast-associated moderate-to-severe TBIs likely result from mechanisms in part similar to those found in non-blast TBI. The degree to which the primary blast wave injures the brain remains controversial [3,4].

Whereas most attention in the Iraq and Afghanistan conflicts initially focused on the moderate-to-severe end of the TBI spectrum, the type of injuries that would be recognized in the field, it soon became apparent that mild TBIs (mTBI) were much more common and were frequently not being recognized at the time of the initial injury [1]. We had previously established conditions that approximate mTBI exposures experimentally. These studies found that exposures up to 74.5 kPa, while representing a blast level that is transmitted to the brain [5], led to no persistent neurological impairments or lung damage [6], although animals subjected to repetitive blast exposure, which has been common in the current conflicts [2], exhibited a variety of chronic behavioral and biochemical changes [7,8]. In contrast, animals exposed to 116.7 kPa blast exposures frequently had gross cerebral and subdural hemorrhages as well as contusions and significant lung pathology [5,6,9], features that are not consistent with mTBI.

In the present study we explored the pathological effects of blast overpressure shock waves in rats exposed to 74.5 kPa blast exposures. We describe a type of shear injury in the brain that has not been described in non-blast TBI models and appears to be unique to blast-associated brain injury.

## Methods

### Animals

All studies were approved by the Institutional Animal Care and Use Committees of the Naval Medical Research Center and the James J. Peters VA Medical Center. Two-month-old male Long Evans Hooded rats (250-350 g; Charles River Laboratories International, Wilmington, MA, USA) were used. Animals were housed at a constant 22°C temperature in rooms on a 12:12 hour light cycle with lights on at 7 AM. All animals were individually housed in standard clear plastic cages equipped with Bed-O’Cobs laboratory animal bedding (The Andersons, Maumee, OH, USA) and EnviroDri nesting paper (Sheppard Specialty Papers, Milford, NJ, USA). Access to food and water was *ad libitum*.

### Blast overpressure exposure

Rats were subjected to overpressure exposure using the Walter Reed Army Institute of Research (WRAIR) shock tube that simulates the effects of air blast exposure under experimental conditions [10]. The shock tube has a 0.32-m circular diameter and is a 5.94 m-long steel tube divided into a 0.76-m compression chamber separated from a 5.18-m expansion chamber. The compression and expansion chambers are separated by polyethylene Mylar™ sheets (Du Pont, Wilmington, DE, USA) that control the peak pressure generated [5,6,10]. The peak pressure at the end of the expansion chamber is determined by piezoresistive gauges specifically designed for pressure–time (impulse) measurements (Model 102M152, PCB, Piezotronics, Depew, NY, USA). This apparatus has been used in several studies to deliver blast overpressure injury to rats [5-9]. Individual rats were anesthetized using an isoflurane anesthesia system consisting of a vaporizer, gas lines and valves, and an activated charcoal scavenging system adapted for use with rodents. Rats were placed into a polycarbonate induction chamber, which was closed and immediately flushed with a 5% isoflurane mixture in air for 2 minutes. Rats were placed into a cone-shaped plastic restraint device and then placed in the shock tube. Movement was further restricted during the blast exposure using a 1.5-cm diameter flattened rubber tourniquet tubing. Three tourniquets were spaced evenly to secure the head region and the upper and lower torso while the animal was in the plastic restraint cone. The end of each tubing was threaded through a toggle and run outside of the exposure cage where it was tied to affix the animal firmly and prevent movement during the blast overpressure exposure without restricting breathing. Rats were randomly assigned to sham or blast conditions with the head facing the blast exposure without any body shielding resulting in a full body exposure to the blast wave. Further details of the physical characteristics of the blast wave have been described elsewhere [6]. Blast-exposed animals received one or three 74.5 kPa exposures. Animals that received three exposures received one exposure per day for three consecutive days. Except for blast exposure, controls were treated identically receiving anesthesia and being placed in the blast tube. For long term studies (four months and over) the animals were transferred to the James J. Peters VA Medical Center within 10 days of blast exposure. The number of animals examined under each condition is shown in Table 1.

**Table 1:** Experimental design

**Group**	**Blast condition**	**Time harvested (post-blast)**	**N Blast**	**N control**
1x-acute	1x74.5 kPa	24 h	5	5
3x-acute	3x74.5 kPa	24 h	5	5
3x-chronic	3x74.5 kPa	4-10 months	17	14

### Histopathological and immunohistochemical analyses

Animals were anesthetized with ketamine (65 mg/kg)/xylazine (13 mg/kg)/acepromazine (2 mg/kg) and transcardially perfused with cold 4% paraformaldehyde in phosphate-buffered saline (PBS). Brains were removed and postfixed overnight in the same fixative. To exclude perfusion artifacts in some cases the brain was dissected and immersion fixed in 4% paraformaldehyde in PBS. Coronal sections of 50 μm thickness were prepared with a Leica VT1000 Vibratome (Vienna, Austria) and stored in sterile PBS at 4°C. For general histopathology serial sections were selected at 500 μm intervals, air-dried and stained with hematoxylin and eosin (H&E).

Immunohistochemical staining was performed on free-floating sections. The primary antibodies used were a rabbit polyclonal anti-collagen IV antiserum (1:500; Millipore, Billerica, MA, USA), a rabbit polyclonal anti-ionized calcium-binding adaptor molecule 1 (Iba-1, 1:400; Wako, Richmond, VA, USA), a rabbit polyclonal anti-laminin (1:150; Sigma-Aldrich, St. Louis, MO, USA), a rabbit polyclonal antibody against the neurofilament heavy subunit (NFH, 1:300, Sigma-Aldrich), a mouse monoclonal antibody against phosphorylated neurofilaments (SMI31, 1:500, Covance Research Products, Denver, PA, USA), a mouse monoclonal antibody against the APP-N-terminal region (clone 22C11, 1:150, Millipore), a mouse monoclonal anti-β-III tubulin (Tuj, 1:500; Covance), a mouse monoclonal anti-2′,3′-cyclic nucleotide 3′-phosphodiesterase (CNPase, 1:200; Millipore), a mouse monoclonal anti-α-smooth muscle actin (α-SMA, 1:500; Sigma), a rat monoclonal anti-glial fibrillary acidic protein (GFAP, 1:500, gift of Dr. Robert Lazzarini), a mouse monoclonal antibody that recognizes tau protein phosphorylated at Ser202 (CP-13, 1:300, gift of Dr. Peter Davies). Sections were blocked with Tris-buffered saline (TBS; 50 mM Tris–HCl, 0.15 M NaCl pH 7.6), and 0.15 M NaCl/0.1% Triton X-100/5% goat serum (TBS-TGS) for 1 hour, and the primary antibody was applied overnight in TBS-TGS at room temperature. Following washing in PBS for 1 hour, immunofluorescence staining was detected by incubation with species-specific AlexaFluor secondary antibody conjugates (1:300; Molecular Probes, Burlingame CA, USA) for 2 hours in TBS-TGS. Nuclei were counterstained with 1 μg/ml 4′,6-diamidino-2-phenylindole (DAPI). Immunoperoxidase staining for collagen IV was performed on pepsin-digested tissue as previously described [11] and sections were counterstained with 0.5% cresyl violet. Stained sections were photographed on a Zeiss AxioImager microscope using the AxioVision Release 4.3 software program (Zeiss, Thornwood, NY, USA), a Nikon Eclipse E400 connected to a DXC-390 CCD camera (Nikon, Melville, NY, USA) or a Zeiss LSM 710 confocal microscope. Unstained sections of fixed brain were photographed on a Nikon SMZ1500 stereomicroscope equipped with an oblique coherent contrast illumination system and connected to a SPOT RT digital camera (Sterling Heights, MI, USA). Digital images were color balanced using Adobe Photoshop 11.0 (Adobe Systems, San Jose, CA, USA).

For TUNEL (terminal deoxynucleotidyltransferase-mediated dUTP nick-end labeling) staining, sections were washed in TBS, permeabilized with 0.1% Triton X-100 in TBS for 1 hr and washed extensively with TBS. End labeling of DNA with fluorescein-dUTP was performed using a commercial kit (Roche, Indianapolis, IN, USA). After several washes with PBS, the sections were blocked and stained with a mouse monoclonal anti-α-smooth muscle actin antibody as described above.

## Results

### Animals groups and study rationale

We have been examining the acute and chronic effects of blast exposure in the rat focusing on conditions that mimic an mTBI exposure [6-8]. A pressure of 74.5 kPa was chosen based on previous studies suggesting that it best approximates an mTBI exposure [6,8]. Multiple blast exposures have been common in the conflicts in Iraq and Afghanistan and Hoge et al. [2] found that more than 50% of soldiers returning from Iraq who reported no injuries still reported at least two episodes in which an IED exploded near the soldier. This figure rose to nearly 90% among soldiers who suffered mTBIs. We therefore included rats that received three 74.5 kPa blast exposures administered on consecutive days. During the course of our prior studies brain tissue was collected at times ranging from 4 to 10 months following blast exposure (3×-chronic exposure). Here we took advantage of the availability of this tissue to examine the histopathological consequences of blast exposure, supplementing it with tissue from rats that received one or three blast exposures and were sacrificed at 24 hours post-blast (1×− and 3×−acute exposure). Table 1 contains a summary of the animals examined. There was no mortality in any of the blast-exposed or control groups.

### Screen for neuropathology

We performed a general screen for neuropathology on all the rats listed in Table 1. Following perfusion, brains were cut into 50 μm-thick Vibratome sections and initially imaged by diascopic bright/dark field microscopy for gross abnormalities. H&E staining was performed on every 10th section from each brain. Based on these observations sections were selected for further analysis.

### Intraventricular hemorrhages are common following blast exposure

**Figure 1:**
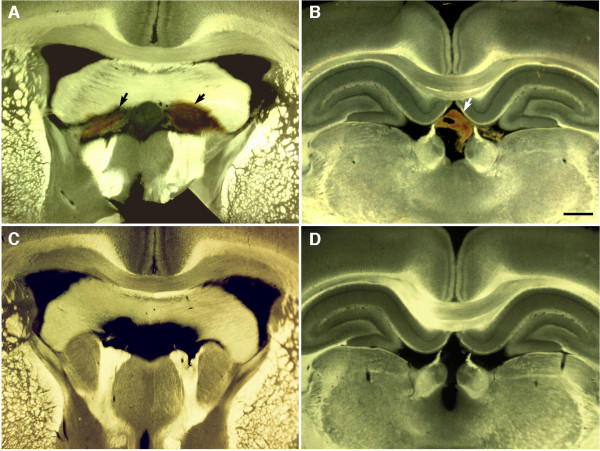
**Intraventricular hemorrhages following blast exposure.** Shown are Vibratome sections imaged by diascopic bright/dark field illumination from 1×-acute blast-exposed animals. Note blood (arrows) in the region of the choroid plexus **(A)** and dorsal third ventricle **(B)**. Panels **(C)** and **(D)** show comparable regions from control brains for **(A)** and **(B)**. Scale bar: 1 mm.

Imaging of sections by diascopic bright/dark field illumination revealed the presence of intraventricular hemorrhage in 40% of the brains examined at 24 hours in rats exposed to either single (2/5 animals) or 3 (2/5 animals) blast exposures (Figure 1). Hemorrhages often appeared associated within the choroid plexus although blood was frequently evident more widely in the ventricles. With the exception of one animal, no hemorrhages were detected in the brain parenchyma.

### Lack of histopathology in blast-exposed brains

A screen for neuropathology did not reveal any widespread histopathologic alterations in H&E-stained sections in either the acute (1×- and 3×-acute) or chronic (3×-chronic) groups. Immunohistochemical staining for neurofilament proteins or β-III tubulin (Tuj) revealed no general neuronal pathology. No axonal pathology was found by immunostaining for the amyloid precursor protein (APP) whose accumulation in axons is widely used as a marker of axonal injury in humans and experimental animal models of TBI [12]. TUNEL staining did not reveal evidence of generalized apoptosis. CNPase immunostaining was performed as a measure of myelin integrity and was normal. No generalized reactive astrocytosis or microglial activation was detected by staining for GFAP or the activated microglia marker Iba-1. Collagen IV immunostaining revealed no vascular pathology. In contrast to a recent study showing accumulation of hyperphosphorylated tau in both human cases and a mouse models of blast associated brain injury [13] we did not detect any accumulation of hyperphosphorylated tau in blast-exposed animals by immunostaining with the antibody CP-13, which recognizes phosphorylated tau at Ser202.

### Blast induces shear-related injuries in brain

**Figure 2:**
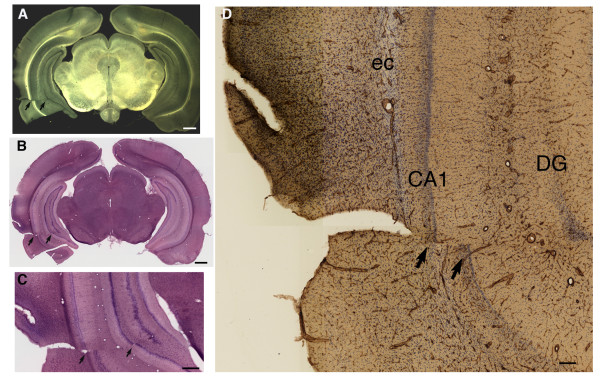
**Blast-induced shear-related injury in the brain.** Shown are sections from a rat sacrificed four months after receiving a 3 × 74.5 kPa blast exposure. Diascopic bright/dark field images **(A)** and H&E stained sections **(B-C)** are shown that demonstrate a discontinuity in the tissue (indicated by arrows in all panels) in contrast to the normal appearance of the contralateral side in panels **(A)** and **(B)**. Panel (**D**) shows a reconstructed image of the area around the lesion immunostained with an anti-collagen IV antibody, which stains mainly blood vessels, and is counterstained with cresyl violet. The external capsule (ec) as well as the CA1 pyramidal cell layer and the dentate gyrus (DG) of the hippocampus are indicated. The discontinuity in the hippocampal pyramidal cell layers is indicated by arrows. Chronic disruption of the perirhinal cortex, external capsule and hippocampus are visible. Scale bars: 1 mm, **A-B**; 150 μm, **C**; 100 μm, **D**.

Despite the lack of any general histopathology, we observed focal lesions in many brains. In animals sacrificed more than 4 months after blast exposure we identified such lesions in 41% (7/17 brains; see Table 2) of blast-exposed animals compared to none in the controls (0/14). An example of such a lesion is shown in Figure 2 in a rat that received 3 × 74.5 kPa blast exposures and was sacrificed 4 months after the last exposure. The lesion was first visible when Vibratome sections were examined under diascopic bright/dark field illumination (arrows in Figure 2A; compare to contralateral side). At first glance, the lesion appeared as a discontinuity in the tissue that might initially be thought to be a sectioning artifact. However, on histological examination it became apparent that the lesion, which resembles a rip or tear, had produced a discontinuity in the tissue causing separation of the layers of the hippocampus proper and dentate gyrus (Figure 2B-C). In fact, as shown in Figure 2D, the fissure spanned the perirhinal cortex, external capsule (ec), hippocampal CA1 and dentate gyrus (DG) resulting in a misalignment of the tissue layers.

**Table 2:** Brain pathology associated with experimental mTBI

**Animal ID**	**Blast condition**	**Time harvested**	**Lesions observed**
B2	1 x 74.5	24 h	Tear causing repositioning of part of the caudate-putamen into the insula.
B3	1 x 74.5	24 h	Blood in dorsal 3^rd^ and lateral ventricles as well as choroid plexus.
B4	1 x 74.5	24 h	Blood in dorsal 3^rd^ and lateral ventricles.
B1	3 x 74.5	24 h	Blood in dorsal 3^rd^ ventricle.
B5	3 x 74.5	24 h	Blood in aqueduct, dorsal 3rd and lateral ventricles; vascular disruption affecting external capsule and CA1 field; blood in the adjacent parenchymal tissue.
542	3 x 74.5	6 months	Tear spanning the perirhinal cortex, external capsule, hippocampal CA1 region and dentate gyrus resulting in tissue architectural abnormalities.
2	3 x 74.5	6 months	Lesion in the secondary auditory cortex expanding to the perirhinal region.
1	3 x 74.5	9 months	Tear at the surface of the secondary somatosensory cortex extending into the insular cortex; lesion involves repositioning of cortical layers; disruption of piriform cortex by the insertion of olfactory tubercle/lateral olfactory tract tissue.
590	3 x 74.5	10 months	Disruption of layers I, II and III in primary visual cortex; presence of ectopic neurons in layer I; lesion of hippocampal CA1.
591	3 x 74.5	10 months	Tear and repositioning of layers I, II and III in primary somatosensory cortex; ectopic neurons in layer I; disruption of parietal and somatosensory cortex and ventral/intermediate entorhinal cortex by the insertion of ectopic tissue.
595	3 x 74.5	10 months	Lesion in motor cortex altering the cortical architecture.
597	3 x 74.5	10 months	Displacement of amygdalohippocampal and posteromedial cortical amygdaloid nucleus tissue disrupting the ventral hippocampal CA1 field.

**Figure 3:**
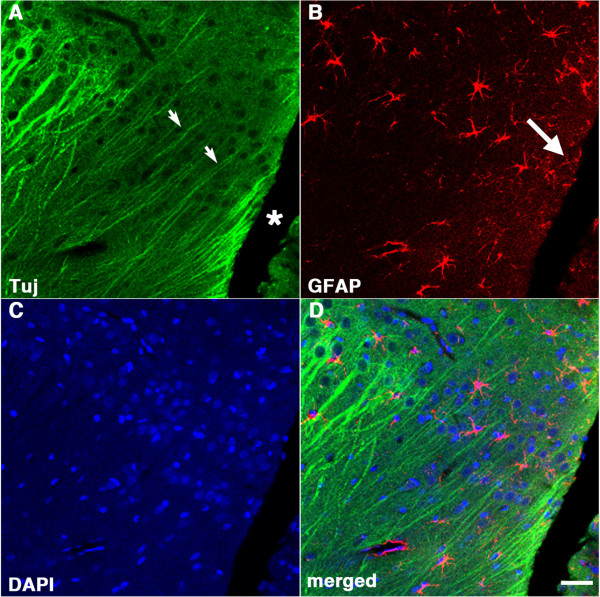
**Gliosis and dendritic changes around blast-induced tear indicate chronicity of the lesion.** Shown is a confocal image at the cortical surface adjacent to the tear (asterisk) illustrated in Figure 2 from a rat sacrificed four months after receiving a 3 × 74.5 kPa blast-exposure. The section was stained with antibodies against β-III tubulin (Tuj, **A**, green), GFAP (**B**, red), and with a DAPI nuclear stain (**C**, blue). A merged image is shown in panel **(D)**. Note the attenuation of the Tuj immunostaining of the dendrites in neurons in cortical layers II and III adjacent to the lesion (arrows in **A**) and the increased GFAP immunostaining adjacent to the lesion (**B**, arrow). Scale bar: 50 μm.

Figure 3 shows staining at the cortical surface around the lesion illustrated in Figure 2 with antibodies against Tuj and GFAP. In the tissue adjacent to the lesion (asterisk in A) thinning of Tuj-immunoreactive dendritic processes is apparent (arrows in A) and increased GFAP immunostaining (arrow in B) is seen at the margins of the lesion indicating that the tear was not an artifact of sectioning but rather a chronic lesion associated with a glial reaction and neuronal injury in the adjacent tissue.

**Figure 4:**
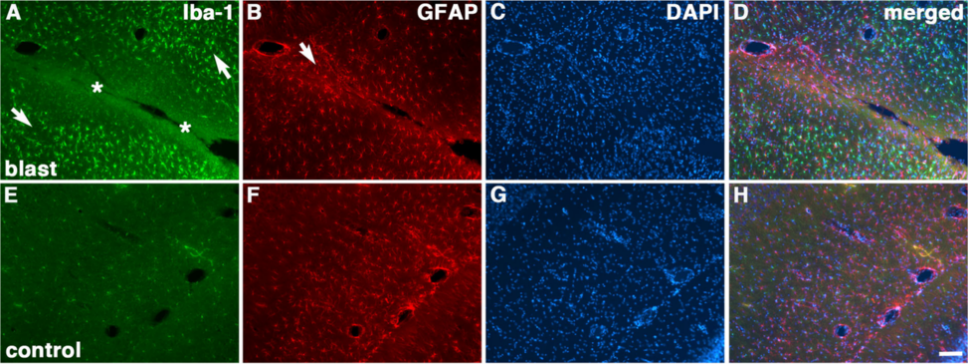
**Microglial activation and gliosis adjacent to a chronic blast-induced tear in the hippocampus.** Brain sections from blast-exposed (3×−chronic sacrificed at 4 months post-blast) and control animals were immunostained with antibodies against Iba-1 (**A** and **E**; microglia, green) and GFAP (**B** and **F**; astrocytes, red). The sections were counterstained with DAPI (**C** and **G**, blue) and merged images are shown in panels **D** and **H**. Upregulation of Iba-1 expression **(A)** is seen in the surrounding hippocampal tissue (white arrows) although not in the region immediately adjacent to the lesion (indicated by asterisks). Increased GFAP staining (**B**, arrow) is seen adjacent to the lesion. Scale bar: 100 μm.

**Figure 5:**
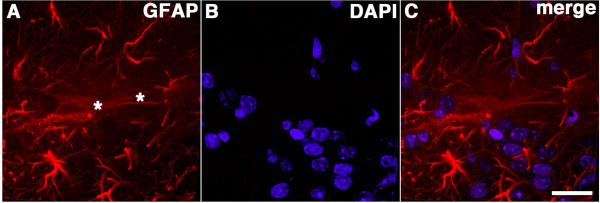
**Gliosis adjacent to a chronic blast-induced lesion.** Shown is confocal imaging of immunostaining for GFAP **(A)** around the tissue tear illustrated in Figure 4. Sections were counterstained with DAPI **(B)** and merged images are shown in panel **C**. Note the concentration of GFAP-immunostained astrocytes around the lesion which is indicated by asterisks. Scale bar: 20 μm.

Figure 4 shows another focal lesion from a 3×-chronic exposure animal that involved the hippocampus. This section was immunostained with antibodies against Iba-1 and GFAP. An increase in GFAP expression was seen at the margins of the lesion (arrow in Figure 4B). Figure 5 shows a higher power confocal image of GFAP immunostaining at the margins of the lesion illustrated in Figure 4. An increase in GFAP immunostainied astrocytes is evident, consistent with chronic gliosis. While the region immediately surrounding the lesion was devoid of Iba-1-expressing cells, there was a dramatic increase in Iba-1 expression in regions adjacent to the tissue tear (arrows in Figure 4A) when compared to the low level of Iba-1 immunostaining normally present in the hippocampus of a control brain (Figure 4E).

**Figure 6:**
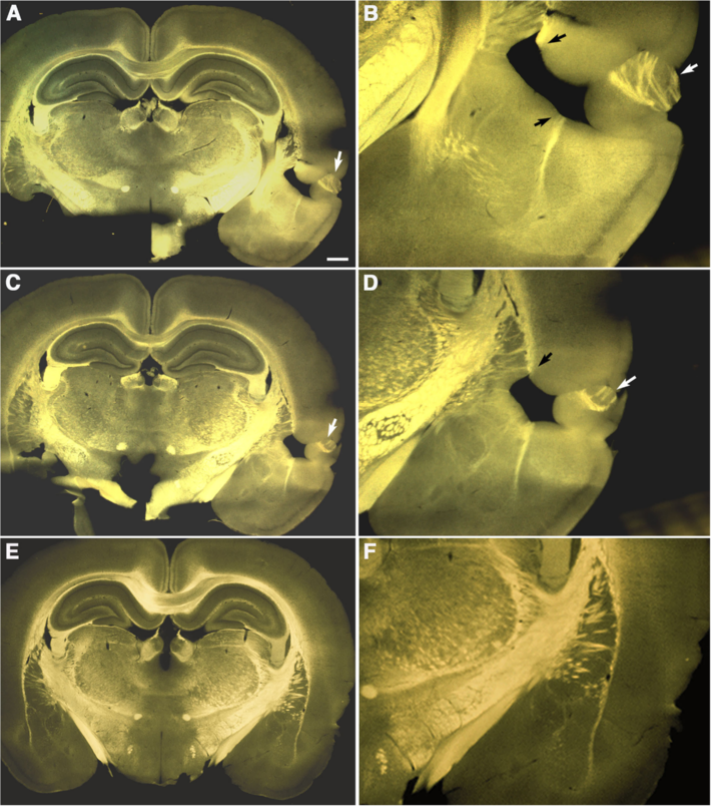
**Mechanical excision and repositioning of a part of the caudate-putamen by the blast.** Shown are diascopic bright/dark field images of Vibratome sections from a 1×–acute exposed rat. Sections in panels **A-B** are 500 μm apart from those in panels **C-D**. White arrows indicate a region of the caudate-putamen that was repositioned into the insular cortex. The lesion also disrupted the external capsule (black arrows in **B** and **D**). Note that the repositioned tissue appears to have been reoriented by 180^o^ compared to its likely original position. Panels **E** and **F** show comparable sections from a control brain. Scale bar: 1 mm, **A**, **C** and **E**; 300 μm, **B**, **D** and **F**.

Similar lesions could be seen in brains examined acutely that sometimes resulted in unusual tissue repositioning leading to dramatic alterations of cerebral architecture. One such example is illustrated in Figure 6, which shows diascopic bright/dark field images from two Vibratome sections that were 500 μm apart taken from a 1×-acute exposure animal. In this example, part of the more rostral caudate-putamen (white arrows in Figure 6A-D), was effectively avulsed by the blast and repositioned into the insular cortex (compare to panels 6E and F from a control brain). This lesion which expanded more than 500 μm in length perpendicular to the sectioning plane resulted in disruption of the external capsule (black arrows in Figure 6B and D).

**Figure 7:**
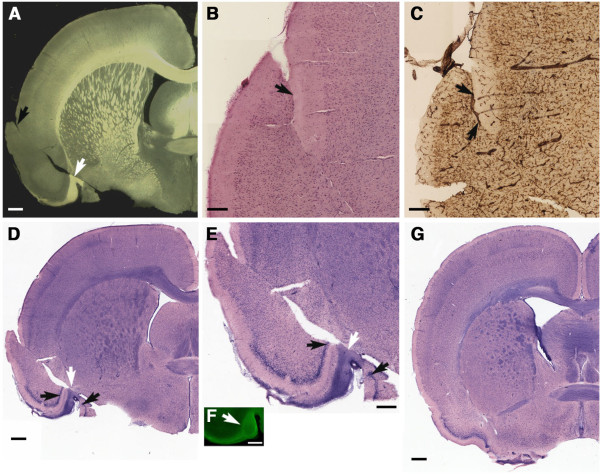
**Blast-induced disruption of the piriform cortex, insular cortex, and secondary somatosensory cortex.** Shown are diascopic bright/dark field images **(A)**, H&E-stained **(B)** and collagen IV-immunostained **(C)** sections from a 3×-chronic exposure animal that was sacrificed 8.5 months post-blast. A tear (black arrow in panel **A**) can be seen that begins at the surface of the secondary somatosensory cortex and extends through insular cortex resulting in misalignment of the insular cortical layers. H&E staining **(B)** of the lesion at the cortical surface shows that layer I (arrow) of the secondary somatosensory cortex has been embedded into layers II and III. In a collagen IV-immunostained section **(C)** adjacent to that shown in panel **B** an artery (black arrows) and two arterioles derived from it are visible. H&E staining **(D-E)** shows interruption of the piriform cortex (black arrows) by the insertion of tissue that appears to have originated in the lateral olfactory tract/olfactory tubercle (white arrow) which is also visible in panel **A** (white arrow). Immunostaining with an antibody against CNPase **(F)** confirmed the myelinated nature of the repositioned tissue. Panel **G** shows the normal appearance of the piriform cortex in an H&E stained section from a control rat. Scale bars: 1 mm, **A**, **D** and **G**; 150 μm, **B-C**; 500 μm, **E-F**.

**Figure 8:**
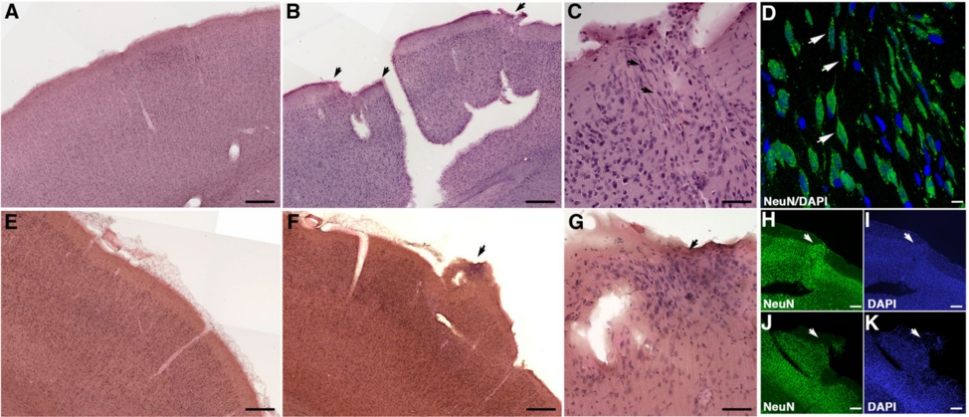
**Ectopic neurons in neocortical layer I of blast-exposed animals.** Shown are H&E (**B** and **C**) or NeuN (**D** and **H**, green) staining of the primary visual cortex from a 3×-chronic exposure animal analyzed 10 months after the blast exposure. H&E staining from the corresponding area in a control rat is shown in panel **A**. Panel **B** shows two areas (arrows) where the cortical layers have been disrupted. In the region indicated by the right arrow, ectopic cells are visible in layer I. This region is shown at higher magnification in panel **C**. The phenotype of the ectopic cells was determined to be neuronal by NeuN immunostaining (**D** and **H**, green), combined with DAPI staining (**D** and **I**, blue). The cortical layers around the left arrows **(B)** are also misaligned. The ectopic neurons were repositioned from cortical layers II and III. Note the presence of spindle-shaped cells (black arrows in **C**) and their NeuN staining shown in **D** (white arrows). Panels **F** and **G** show H&E staining of primary somatosensory cortex from another 3 ×-chronic exposure animal also analyzed at 10 months post blast. Panel **G** shows the same region at a higher magnification. The blast produced a tear that disrupted layers I to III with the result that a portion of layers II and III (arrow in both panels **F** and **G**) was avulsed and repositioned. NeuN **(J)** and DAPI **(K)** staining of the region demonstrated the neuronal character of the ectopic cells. The corresponding area from a control rat is shown in panel **E**. Scale bars: 200 μm, **A**, **B**, **E** and **F**; 50 μm, **C** and **G**; 10 μm, **D**; 100 μm, **H**, **I**, **J** and **K**.

**Figure 9:**
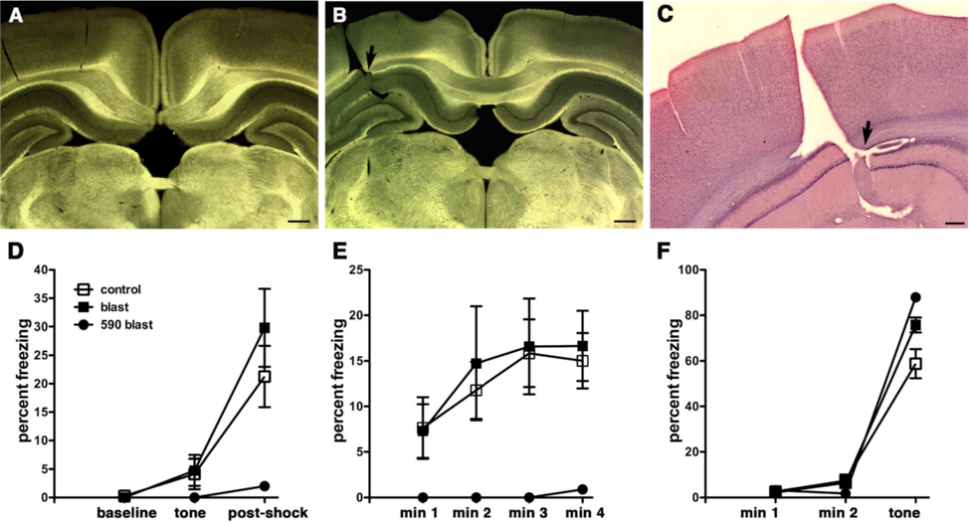
**Disruption in the hippocampus of the CA1 layer by excised lacunosum-moleculare tissue.** Shown are diascopic bright/dark field images from a control **(A)** and a 3×−chronic exposure rat **(B)** analyzed at 10 months post blast (animal 590). A tear in the primary visual cortex is indicated by arrows in panels **B** and **C**. In panel **C**, an H&E stained section is shown in which a fragment from the hippocampal lacunosum-moleculare is visible (arrow in panel **B**) that has been excised and disrupts the hippocampal CA1 field. Panels **D-F** show the response of this rat which was tested in a previously published behavioral study [8] in a contextual and cued fear paradigm. Methods and original pooled group data were described in Elder et al. [8]. Panel **D** shows response during the training phase in which rats were exposed to an 80dB tone that was paired with a foot shock. Freezing behavior was measured at baseline, after the tone and after the shock. Panel **D** shows the test for contextual fear memory performed 24 h after the initial training. Freezing was measured during four minutes in the initial conditioning chamber. Panel **F** shows the cued fear response performed 24 h after the testing in **E**. Animals were placed in a novel context and freezing was measured at baseline and after exposure to the conditioned stimulus (80-dB tone). As compared to the control group (n = 13) and the pooled blast exposed group (n = 14), rat 590 failed to freeze following the pairing of the tone and the foot shock in the conditioning phase. It also showed no freezing in the contextual phase but normal freezing in response to the tone in the cued phase. Scale bars: 1 mm, **A** and **B**; 200 μm, **C**.

Other examples of focal structural alterations are shown in Figures 7, 8 and 9. Figure 7 shows a tear at the surface of the secondary somatosensory cortex in a 3×-chronic exposure rat. The lesion extended through the insular cortex altering the alignment of the cortical layers of the insula with the affected layers interrupted by the tear repositioned in relationship to the outer cortical layers (Figures 7B-C). Collagen IV immunostaining showed the repositioning of a normal appearing artery and two arterioles derived from the parent vessel into the brain parenchyma (Figure 7C). Additional collagen IV immunostaining around the most superficial portion of the tear showed that no vessels or vascular remnants were associated with the tear itself (not shown). At the ventral surface of the brain the lesion resulted in the insertion of a portion of the olfactory tubercle/lateral olfactory tract (white arrow in Figure 7D and E) into the piriform cortex (black arrows; compare to Figure 7G, showing the same region from a control rat). The myelinated nature of the repositioned tissue was confirmed by staining for CNPase (white arrow in Figure 7F).

Disruption of the upper cortical layers I, II, and III in the primary visual cortex in a 3×-chronic exposure animal is illustrated in Figure 8B. In this example two areas are visible where the cortical layers have been disrupted (compare to a control brain in Figure 8A). In the area on the left of the tear in Figure 8B the cortical layers are misaligned. In the region on the right of the tear in Figure 8B ectopic cells are visible in layer I including a variety of spindle-shaped cells (Figure 8C). These cells were identified as neurons based on their immunostaining for NeuN (Figure 8D and H). In another 3×-chronic exposure animal (Figure 8F, G, J and K) a lesion in the primary somatosensory cortex produced a tear that disrupted layers I to III resulting in a portion of layers II and III being avulsed relative to layer I (arrows in Figure 8F, G, J and K; compare to panel E from control brain). NeuN immunostaining (Figure 8J) demonstrated the neuronal character of these ectopic cells, which also included many spindle-shaped neurons. Figures 9B and C show disruption of the CA1 field of the hippocampus by a tear (compare panel 9B to 9A that shows the same region in a control brain). This tear disrupted the primary visual cortex and severely damaged the hippocampal layers (arrow in panel C).

### Behavioral alterations associated with blast-related focal lesions

**Figure 10:**
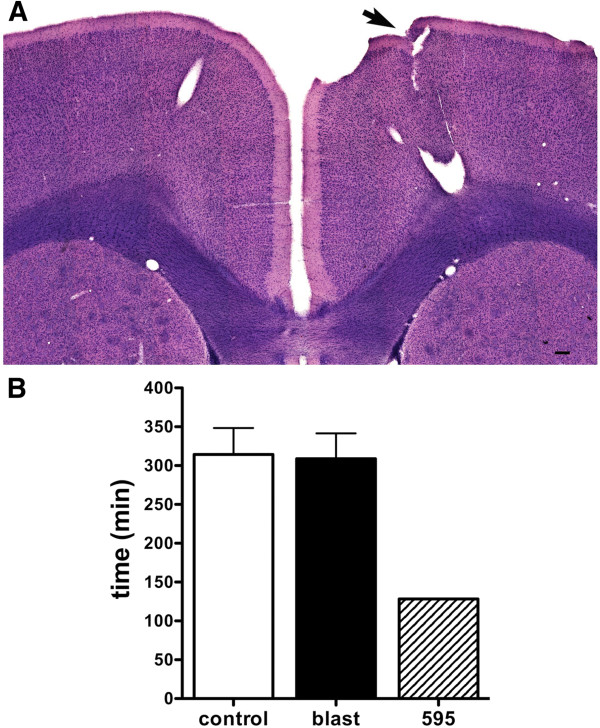
**Reduced center time in open field testing of a rat with a blast-induced lesion in the motor cortex.** Shown is an H&E-stained section **(A)** from a 3×−chronic exposure rat. An arrow points to a lesion in the motor cortex (compare to the unaffected contralateral side). Panel **B** shows center time in an open field test of this animal (rat 595) compared to blast-exposed and control groups (error bars indicate S.E.M.). Methods and original pooled group data were described in Elder et al. [8]. Scale bar: 200 μm.

**Figure 11:**
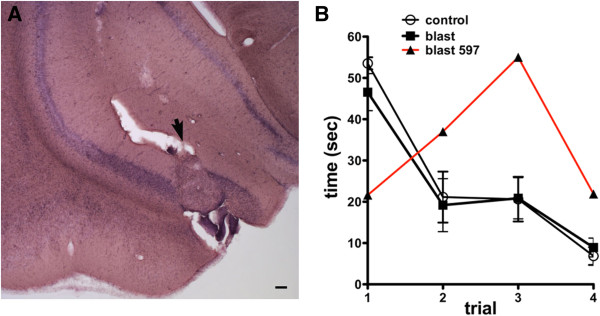
**Association of disruption of the caudoventral hippocampal CA1 field with deficits in spatial learning.** Panel **A** shows an H&E stained section from a 3×−chronic exposure rat with a blast-induced lesion in which a portion of the posteromedial cortical amygdaloid nucleus and posteromedial amygdalohippocampal area has been repositioned into the hippocampal CA1 region. Panel **B** shows performance of this rat (597) in a Morris water maze revealing spatial learning impairments compared to the pooled blast-exposed and control groups (± S.E.M.). Methods and original pooled group data were described in Elder et al. [8]. Scale bar: 200 μm.

We have previously shown that rats exposed to 3 × 74.5 kPa blast exposures exhibit a variety of post-traumatic stress disorder-related traits [8]. Some of the 3×-chronic exposure animals (6 control and 6 blast-exposed) examined here were part of this behavioral study [8]. Due to the heterogeneity in the observed blast-induced brain lesions, we did not expect that a common anatomical lesion would be found that could account for the PTSD-like behavioral phenotype. However, some blast-exposed animals demonstrated deficits in specific behavioral tests. For example the rat in Figure 9 with a lesion of the dorsal hippocampal CA1 exhibited a very unusual response in cued and contextual fear testing. This rat failed to freeze following the pairing of the tone and the foot shock in the conditioning phase and showed no freezing in the contextual phase. Yet it froze normally in response to the tone in the cued phase of testing (Figure 9D-F). Other examples of behavioral abnormalities in rats with blast-related focal lesions are shown in Figures 10 and 11. A rat with a lesion in the motor cortex spent less time in the center of an open field (Figure 10). Figure 11 shows a lesion that disrupts the posterior ventral hippocampal CA1 causing an avulsion of the posteromedial cortical amygdaloid nucleus and posteromedial amygdalohippocampal area. This animal showed deficits in the Morris water maze a hippocampus-dependent task of spatial navigation in contrast to the blast-exposed animals as a group whose behavior was similar to controls. While it is difficult to with confidence ascribe any of these behavioral deficits to the observed lesions, it is clear that some blast-exposed animals with lesions were severely affected in specific behavioral tests.

### Blast-related rips and tears frequently follow vascular fault lines

**Figure 12:**
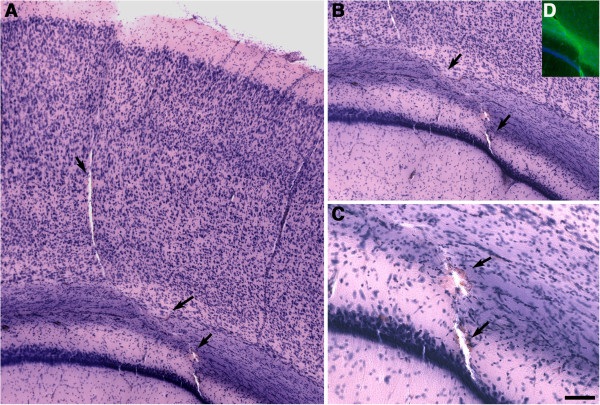
**Blast-induced lesion that follows a vascular fault line and is associated with microscopic evidence of hemorrhage.** Shown are H&E-stained sections **(A-C)** from the brain of a 3×−acute exposure animal. A blast-induced discontinuity in the cortical layers is apparent at the junction between layers I and II that follows the fault line of a penetrating cortical vessel (arrows) in the auditory cortex **(A)** disrupting the external capsule and extending into the hippocampus **(B)**. An area of hemorrhage with parenchymal infiltration of erythrocytes (arrows) is visible in panel **(C)**. Disruption of myelinated fibers in the external capsule was confirmed by immunohistochemical staining with an anti-CNPase antibody **(D)**. Scale bar: 200 μm, **A**, **B**; 100 μm, **C**; 400 μm, **D**.

The lesions often appeared to follow fault lines that seemed to parallel penetrating blood vessels such as those illustrated in Figures 2, 4, 5, and 9. Supporting a vascular origin for some acute lesions, red blood cells could be found within some lesions. For example, Figure 12 shows a lesion involving the auditory cortex in a 3×–acute exposure animal. This lesion also disrupts the external capsule extending into the hippocampus, and red blood cells were visible within the portion passing through the external capsule and CA1.

**Figure 13:**
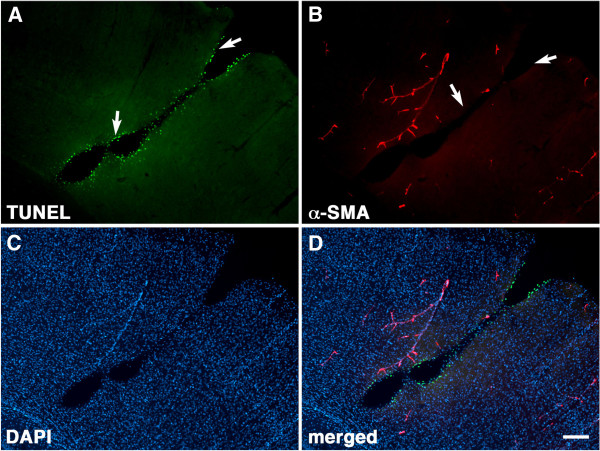
**Apoptotic cells line the margins of a blast-induced cortical tear without evidence of a vascular remnant.** A section from a 3×−acute exposure rat was labeled for TUNEL **(A)**, immunostained for α-smooth muscle actin (**B**, α-SMA) and counterstained with DAPI **(C)**. Merged images are shown in panel **(D)**. A tear lined by apoptotic cells that resembles a vessel is visible in panel **(A)**. However while TUNEL-positive cells (arrows in panel **A**) are seen at the margins of the tear, no staining for α-SMA is apparent at the edges of the lesion (arrows in panel **B**). Scale bar: 200 μm.

However, most lesions could not be unequivocally associated with a vascular origin. For example a tear resembling a penetrating cortical blood vessel from a 3×-acute exposure rat is shown in Figure 13. While the margins of the lesion were lined with apoptotic TUNEL-positive cells, they did not show immunostaining for α-SMA suggesting that there was no vascular remnant and thus that the lesion does not follow a blood vessel.

## Discussion

Whether primary blast forces directly damage the brain is still controversial and if they do, the exact mechanisms that mediate injury remain unknown [3,4,14]. While it was once thought that the skull forms a protective barrier preventing the blast pressure wave from directly damaging the brain [15], studies in animal models subsequently showed that the blast pressure wave is transmitted to the brain with little attenuation [5,13,15-23].

Here, we analyzed the early (24 hours) and long-term (>4 months) pathological effects in the brains of rats exposed to blast overpressure, using a model that approximates a mild TBI exposure. The earliest and most common pathological finding at 24 hours post-blast was the presence of blood in the choroid plexus, ventricles and cerebral aqueduct, occurring even after a single blast exposure. This pathology seems best explained by direct effects of blast on the choroid plexus leading to vascular rupture and blood leakage into the ventricles. These results are in agreement with a previous study indicating that the choroid plexus is extremely sensitive to the blast wave [24].

We did not observe any generalized neuropathology or evidence for diffuse axonal injury as judged by APP immunostaining. We also did not observe accumulation of hyperphosphorylated tau as has been reported in another model of blast TBI [13]. Rather, the most prominent effects were what we describe as focal rips or tears in the tissue. These lesions seem best described as shear-related because they result in displacement of adjacent tissue planes causing a realignment of the layers that in some cases led to avulsion and relocation of tissue. Because the lesions are found at 24 hours post-blast exposure, they appear to represent acute lesions. With time these lesions evolve into chronic lesions that exhibit a glial and microglial reaction as well as a neuronal reaction which includes thinning of dendrites in the adjacent tissue. These results are in agreement with a previous study reporting that blast exposure in rats induces microglial activation and hypertrophy in the brain [25]. The spindle-shaped neurons with elongated nuclei that were observed in some lesions have been described in a previous study in which it was suggested that overpressure shock waves cause the long axis of the neurons to align toward the shock wave source [26].

Interestingly, we found that tears often seemed to follow penetrating cortical vessels suggesting that blood vessels could represent fault lines along which the blast pressure may propagate. Several mechanisms could be envisioned as to how this might occur. In what has been called a thoracic mechanism [3,27], it has been proposed that a high-pressure blast hitting the body can induce oscillating high-pressure waves that can be transmitted through the systemic circulation to the brain. Blood pressure in the systemic circulation has been shown to rise during passage of the blast pressure wave [28-30]. Because the arterial capacity to expand in response to the sudden increase in blood pressure depends in part on the pressure in the surrounding parenchyma, brain damage might result from pressure differentials between the pressure on the arterial walls and that in the neighboring parenchyma. A lower pressure in the surrounding brain would allow the arterial wall to expand as a consequence of a sudden increase in blood pressure leading to tissue damage at high/low pressure interphases. This situation could occur if the blast-induced brain compression is not uniform or if the head is partially exposed to the blast creating regions of higher and lower pressures. The contribution of a thoracic mechanism could be directly tested in our model by performing shielding experiments that limit the blast exposure to either head or body. Simultaneous monitoring of blood pressure and intracranial pressure with comparisons of the time course of intracranial pressure and blood pressure changes could also help to tease out the relationship between systemic and brain factors.

A vascular mechanism is supported by the finding in some instances of microscopic hemorrhages in vessels within the lesion. In other instances, even when a direct vascular lesion is not visible, it can be speculated that lesions followed a penetrating vessel or were the result of pressure transmitted through a specific vascular territory. For example, the lesion in Figure 7 might have arisen from a high-pressure wave transmitted through the vessels supplying the piriform cortex and the lateral olfactory tract resulting in compression and mechanical disruption of the neighboring tissue and causing a tear through which the lateral olfactory tract was avulsed. Dilated vessels such as those seen in Figure 9 in the hippocampus could have been responsible for rupturing and displacing neighboring tissue to the more dorsal CA1 region. However, despite the fact that isolated vascular pathology was observed, few lesions whether acute or chronic showed any evidence of hemorrhage and most lesions could not be unequivocally associated with a vascular origin. In addition, if pressures were transmitted through the vascular bed it is curious that vessels sufficiently dilated to produce the type of lesions observed would not result in more cases of obvious hemorrhage.

Alternatively, hemorrhages might not occur if the blast pressure were transmitted through the vascular compartment but not intravascularly. This could occur if the main pressure wave was being transmitted through the Virchow-Robin compartment. Several studies have documented that intracranial pressure increases acutely following blast exposure [7,15,20-27,31,32]. Increased CSF pressure transmitted through the Virchow-Robin compartment could generate local pressure differentials at the interface between the vascular basal lamina and the surrounding tissues. Shearing along this plane would conceptually leave the blood vessel wall intact preventing hemorrhages.

Computer modeling has suggested that blast-associated shear strains should be at their highest at the brain/CSF interface [33] where cavitation effects could occur which have long been speculated as playing a major role in the deleterious effects of blast exposure [31,34]. Another study suggested that highest shear strains should occur at the skull/brain interface [32] consistent with our observation that lesions are found at the cortical surface where the shear wave would move perpendicular to the longitudinal blast wave. Whether propagating as a pressure wave through the ventricular system or generated at the cortical surface such a mechanism could explain expansion of lesions along vascular fault lines without production of hemorrhage.

## Conclusions

Here we used the rat to model mTBI resulting from blast overpressure exposure. We describe a new type of shear injury in the brain that has not been described in non-blast TBI models and appears to be unique to blast-associated brain injury. Why they occur in such a focal fashion when the entire brain is presumably subjected to the same blast exposure remains unclear. Yet, the fact that lesions often follow penetrating cortical vessels suggests that blood vessels may represent the fault lines along which the most damaging blast pressure is transmitted.

Functionally, the effects of these lesions remain speculative. In prior studies we have shown that these animals exhibit chronic behavioral and biochemical changes [7,8]. Focal lesions might be responsible for focal dysfunction in some animals. However, given the low occurrence of the lesions in a given animal, it is unclear whether they can explain the full behavioral and biochemical phenotype. However, the dramatic nature of the lesions, from a neuropathological point of view, suggests that locally constrained but significant parenchymal stress and damage may result in more widespread functional consequences in our model.

## Abbreviations

APP: Amyloid precursor protein; CNPase: 2′,3′-cyclic-nucleotide 3′-phosphodiesterase; DAPI: 4′,6-diamidino-2-phenylindole; GFAP: Glial fibrillary acidic protein; Iba-1: Ionized calcium-binding adapter molecule 1; IED: Improvised explosive devices; kPa: kilopascal; mTBI: mild TBI; PBS: Phosphate-buffered saline; PTSD: Post-traumatic stress disorder; SMA: Smooth muscle actin; TBI: Traumatic brain injury; TBS: Tris-buffered saline; TUNEL: Terminal deoxynucleotidyltransferase-mediated dUTP, nick-end labeling.

## Competing interests

The authors declare that they have no competing interests.

## Authors’ contributions

MAGS: design of the experiments, analysis and interpretation of the data, execution of neuropathological characterization and manuscript writing; RDG: design of the experiments, analysis of the data, execution of neuropathological characterization and manuscript writing; AJP and PEP: execution of neuropathological characterization; MCS: execution of blast exposure experiments; EMJ and AAH: execution of blast exposure experiments; WGMJ confocal imaging; FJY: neuropathological characterization; DLD: neuropathological characterization; NPD: behavioral testing; RMC: design of blast experiments; MC: design of blast experiments; PRH: important intellectual content to the experimental design, data analysis and interpretation and manuscript writing; STA: design of blast experiments, interpretation of data and manuscript writing; GAE: design of the study, analysis and interpretation of the data, manuscript writing; All authors read and approved the final manuscript.
